# *De-novo* assembly of mango fruit peel transcriptome reveals mechanisms of mango response to hot water treatment

**DOI:** 10.1186/1471-2164-15-957

**Published:** 2014-11-05

**Authors:** Neta Luria, Noa Sela, Mor Yaari, Oleg Feygenberg, Ilana Kobiler, Amnon Lers, Dov Prusky

**Affiliations:** Department of Postharvest Science of Fresh Produce, ARO, the Volcani Center, Bet Dagan, 50250 Israel; Department of Plant Pathology and Weed Research, ARO, the Volcani Center, Bet Dagan, 50250 Israel

**Keywords:** *Mangifera indica*, Transcription profiling, RNA-seq, Induced resistance, Sugar metabolism, Lenticel discoloration, Chlorophyll metabolism, Postharvest diseases

## Abstract

**Background:**

The mango belongs to the genus *Mangifera*, consisting of numerous tropical fruiting trees in the flowering plant family, Anacardiaceae. Postharvest treatment by hot water brushing (HWB) for 15–20 s was introduced commercially to improve fruit quality and reduce postharvest disease. This treatment enabled successful storage for 3–4 weeks at 12°C, with improved color and reduced disease development, but it enhanced lenticel discoloration on the fruit peel. We investigated global gene expression induced in fruit peel by HWB treatment, and identified key genes involved in mechanisms potentially associated with fruit resistance to pathogens, peel color improvement, and development of lenticel discoloration; this might explain the fruit’s phenotypic responses.

**Results:**

The mango transcriptome assembly was created and characterized by application of RNA-seq to fruit-peel samples. RNA-seq-based gene-expression profiling identified three main groups of genes associated with HWB treatment: 1) genes involved with biotic and abiotic stress responses and pathogen-defense mechanisms, which were highly expressed; 2) genes associated with chlorophyll degradation and photosynthesis, which showed transient and low expression; and 3) genes involved with sugar and flavonoid metabolism, which were highly expressed.

**Conclusions:**

We describe a new transcriptome of mango fruit peel of cultivar Shelly. The existence of three main groups of genes that were differentially expressed following HWB treatment suggests a molecular basis for the biochemical and physiological consequences of the postharvest HWB treatment, including resistance to pathogens, improved color development, and occurrence of lenticel discoloration.

**Electronic supplementary material:**

The online version of this article (doi:10.1186/1471-2164-15-957) contains supplementary material, which is available to authorized users.

## Background

Mango (*Mangifera indica*) belongs to the plant family Anacardiaceae, which includes numerous tropical fruiting trees; it is native to South Asia, from where it has been distributed worldwide to become one of the most cultivated fruits in the tropics, with significant economic importance [1, 2]. High-quality fruits should be free of external damage, bruises, latex or sap injury, and decay. The storage life of mangoes is limited to 3 or 4 weeks at 10 - 15°C [1, 2], but production and postharvest practices, as well as novel technologies and packinghouse management, contribute greatly to retention of the fruit’s external quality throughout the worldwide supply chain [2, 3].

Mango losses after harvest are caused by: harvesting at inappropriate stages of fruit maturity, mechanical damage during harvesting or through improper field handling, sap burn, discoloration of lenticels, fruit softening, chilling injury, and/or disease development and pest damage [4–8]. Two main factors affecting fruit quality are lenticel discoloration and postharvest disease [9].

Lenticel discoloration is a superficial blemish that affects some cultivars, imparting a speckled appearance to the fruits, which then are regarded as less desirable and are downgraded, although the speckled appearance does not affect fruit internal quality. Blemish development is limited to the lenticel perimeter and the immediately adjacent area, and does not extend deeper than the outermost layers of the rind [10].

Postharvest diseases reduce fruit quality and result in severe losses [11]. In Israel, the main postharvest disease is alternaria black spot (ABS), caused by the fungus *Alternaria alternata*, which penetrates the fruit during its growth and is affected by the relative humidity in the orchard [12, 13]; following penetration, the fungus remains quiescent until fruit harvest and ripening [14, 15]. Stem-end rots that occur following long periods of storage are caused in Israel, mainly by *Phomopsis mangiferae* [12, 16].

Control of postharvest development of side rots caused by *A. alternata*, and stem-end rot caused by *P. mangiferae*, has been achieved by adopting a series of postharvest management practices, including hot-water brushing (HWB) for 15–20 s [2] and, in some cases, application of acid prochloraz and waxing with a polyethylene emulsion [2]. This treatment enables commercially successful storage for 3–4 weeks at 12°C and ripening for an additional week at 20°C.

We recently demonstrated that HWB stresses the fruits and activates processes that could reduce fruit quality after storage [9]. The objective of the present study was to use gene-expression profiling to elucidate the biological mechanisms activated in mango by HWB and that mediate fruit quality and resistance to postharvest diseases. Mango fruits of cultivar Shelly were subjected to a commercial HWB procedure followed by a detailed transcriptomic analysis that used next-generation sequencing platforms. The differential gene-expression profiles of treated fruits indicated several transient HWB-regulated mechanisms, including: expression of host-resistance to pathogens related genes; transient decrease in the expression of chlorophyll catabolism- and photosynthesis; and a late decrease in the expression of genes that modulate processes related to glucose and flavonoid metabolism. The present data suggest fine control of the fruit response by the HWB exogenous treatment associated with packinghouse handling that may strongly modulate fruit quality during storage.

## Results and discussion

### Characterization of mango transcriptome assembly

**Figure 1:**
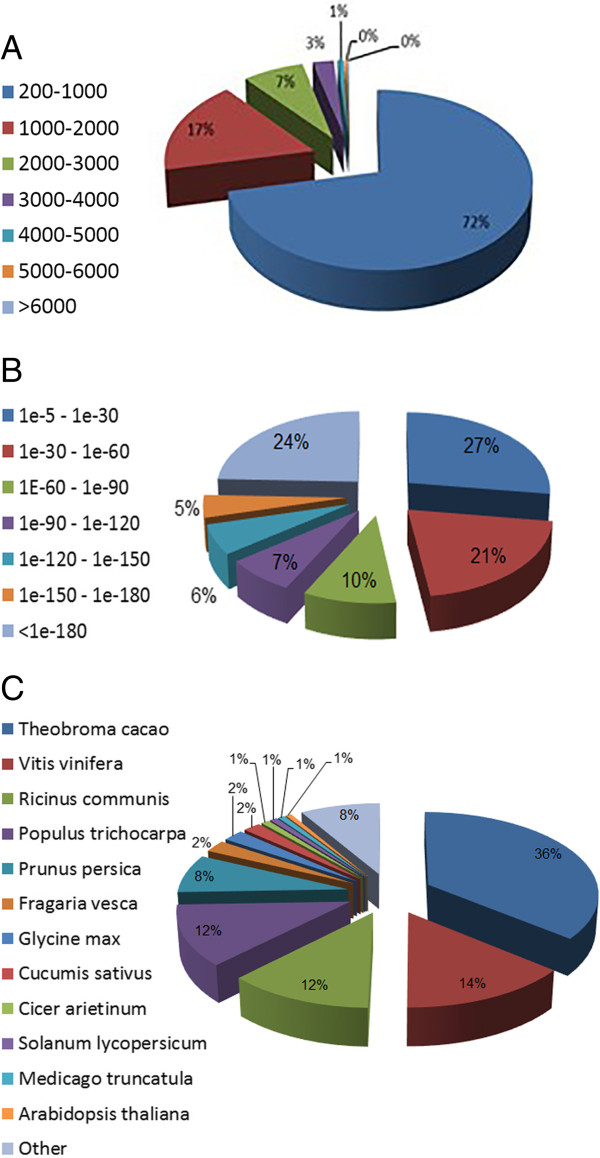
**Characterization of de-novo assembly of mango transcriptome.** All distinct gene sequences that had BLAST annotations within the non-redundant protein database with a cut-off E-value ≤10^-5^ were analyzed for: **(A)** transcript length; **(B)** E-value distribution; and **(C)** species distribution.

**Figure 2:**
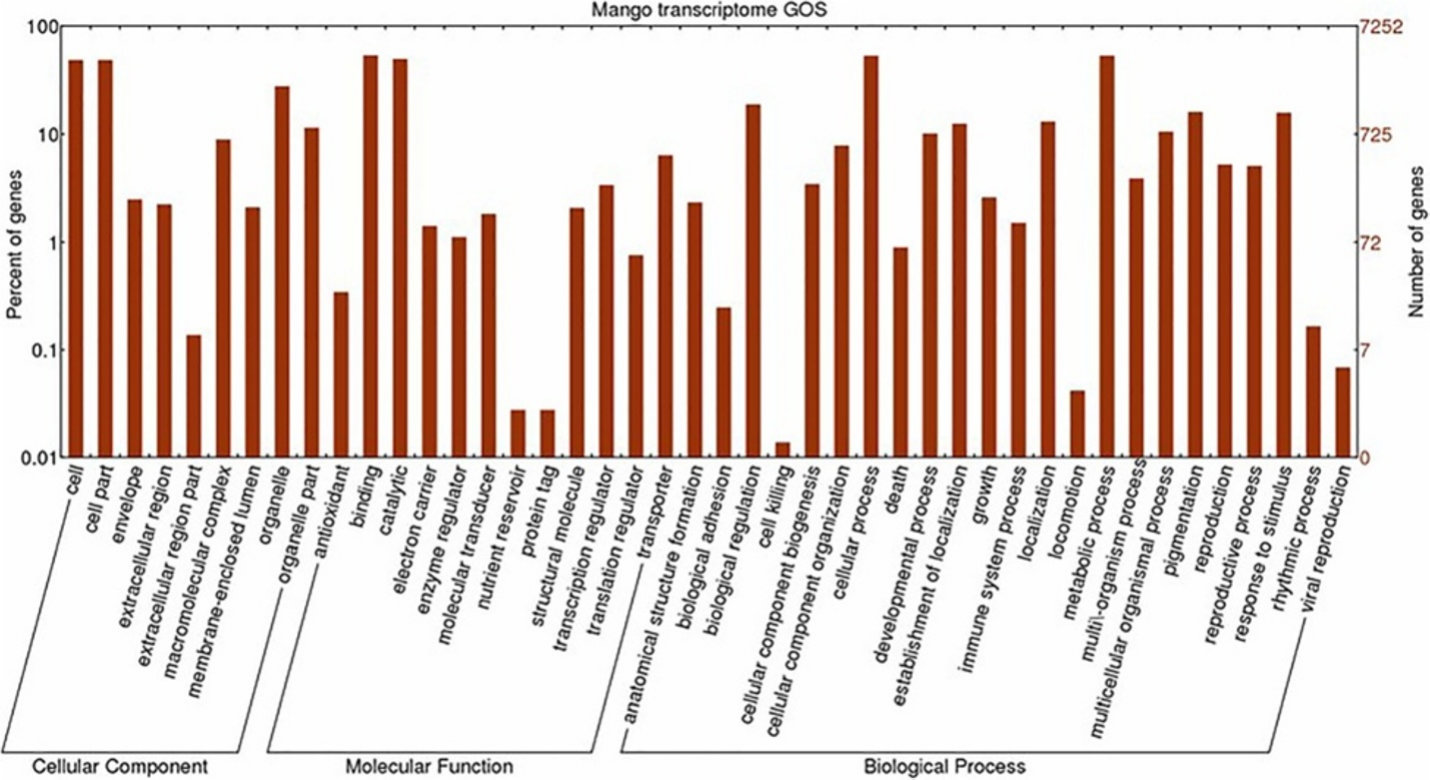
**Gene ontology (GO) classification of the** ***Mangifera indica*** **transcripts.** Out of 57,544 transcripts, 28,317 sequences were annotated within the GO database into three main categories: cellular component, molecular function, and biological process. The y-axis indicates the number of transcripts; the x-axis indicates the GO category.

The mango is a member of the family Anacardiaceae and is an allotetraploid (2n =40) fruit tree with a small genome size of about 450 Mbp [17]. A new mango transcriptome was assembled from 8.6-Gbp sequence data (coverage of 190-fold) by using Trinity [18] software, which generated 57,544 contigs with N50 of 1,598 bases and an average length of 863.3 bases (Figure 1A). To identify the putative functions of assembled transcripts, a sequence-similarity search was conducted against the NCBI non-redundant (NR) database by using a BLASTx search with a cut-off E value of 10^-5^. A total of 35,719 transcripts (62.07%) showed significant similarity to known proteins in the NR database. Based on the NR annotations, 53.12% of the annotated sequences showed very high homology (E-value <10^-60^), and 20.9% showed high homology (10^-60^ < E-value <10^-30^). An additional 25.9% showed homology (10^-30^ < E-value <10^-5^) to available plant sequences (Figure 1B). With respect to species, 35.9 and 14.2% of the unique sequences had top matches to sequences from *Theobroma cacao* and *Vitis vinifera*, respectively, with additional hits to *Ricinus communis* (12.1%), *Populus trichocarpa* (12%), *Prunus persica* (7.5%), *Fragaria vesca* (2.3%(, *Glycine max* (2.2%) and *Cucumis sativus* (1.8%) (Figure 1C). Gene ontology (GO) [19] was used to classify the functions of the predicted mango genes. Based on sequence homology, a total of 28,317 transcripts (49.2%) could be categorized into one of three main categories: biological process, cellular component, and molecular function (Figure 2).

### Profiling the expression of mango genes following HWB treatment

The variation in gene-expression profiles in mango fruit harvested at the mature-green stage was analyzed comparing the gene expression immediately after HWB (10–20 min considered as time 0), and at 4, 17 and 48 h after the treatment with those of untreated fruits at the same time points. Both HWB and control fruits were stored at 12°C. Two basic criteria were used to define differential gene expression: a twofold difference in transcript levels between treated and control fruits, and a *P*-value <0.05 after false-discovery-rate (FDR) determination (Additional file 1: Table S1, Additional file 2: Table S2, Additional file 3: Table S3, Additional file 4: Table S4).

**Figure 3:**
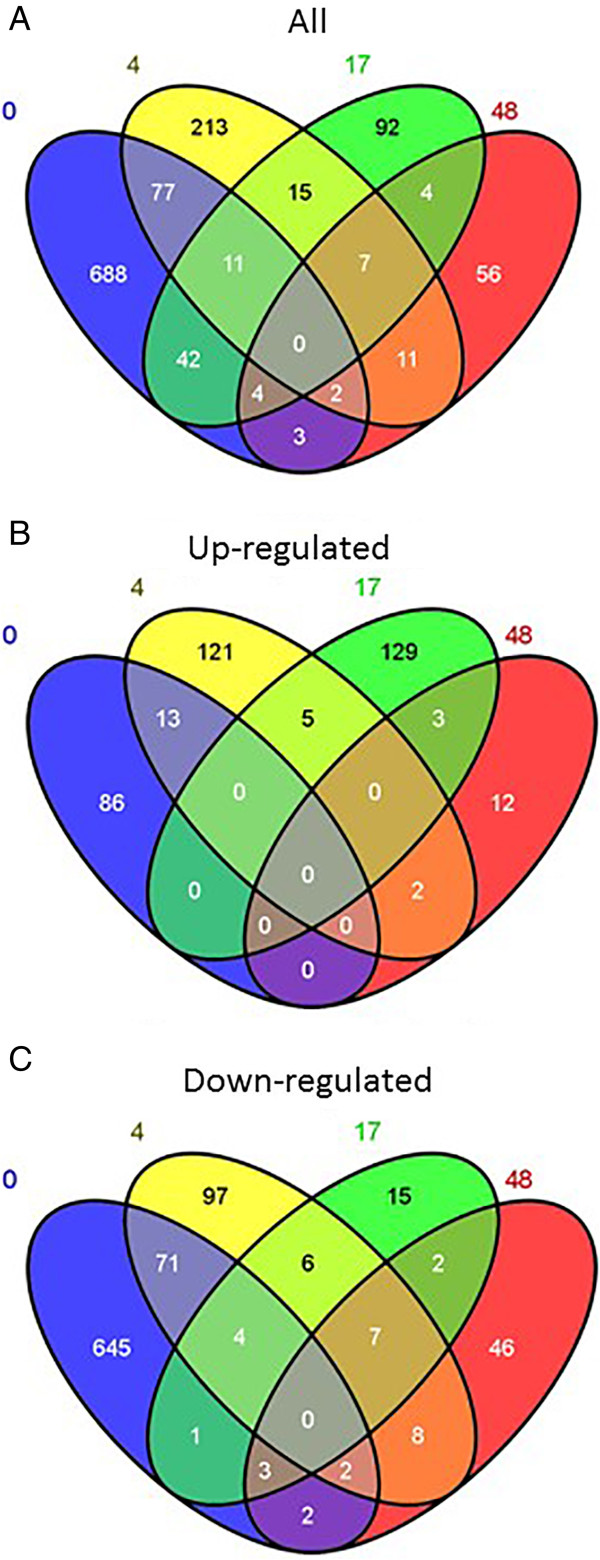
**Venn diagram showing number of overlapping and non-overlapping differentially expressed mango fruit genes at different sampling times after HWB treatment. (A)** All differentially expressed genes; **(B)** differentially expressed genes that were upregulated; **(C)** differentially expressed genes that were downregulated. Fruit tissue was sampled at 0, 4, 17 and 48 h after the HWB treatment, and compared with that of untreated fruits sampled at the same time points.

Analysis of gene responses revealed a decrease in the number of differentially expressed genes from time 0 to 48 h: immediately after HWB treatment (10-20 min was considered as 0 h) 827 genes were differentially expressed whereas 48 h later only 87 genes showed differential expression (Table 1). Venn diagrams (Figure 3) of the differential expression at the four different sampling times showed that most of differentially expressed genes are unique to one of the time points.

**Figure 4:**
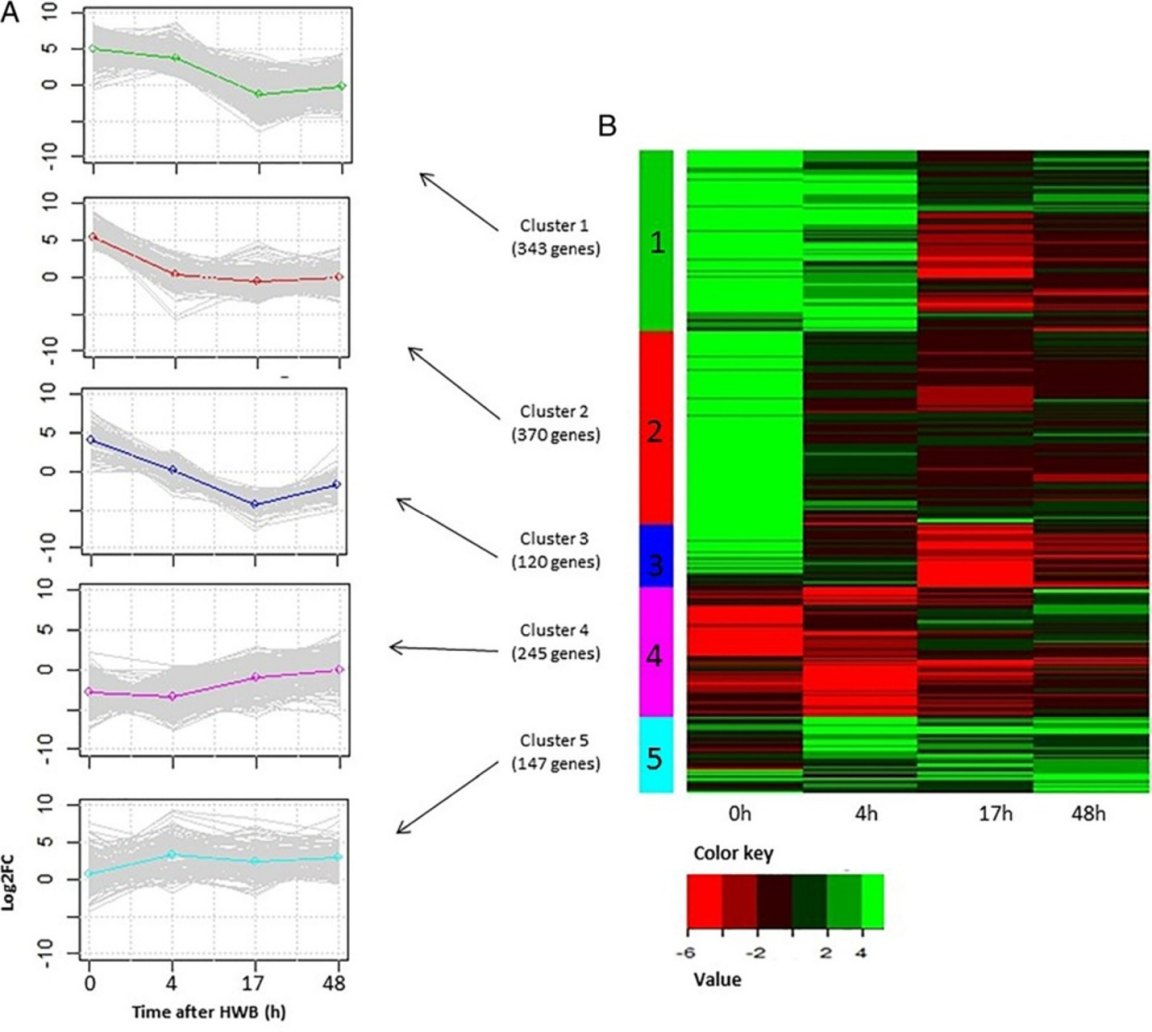
**Heat-map diagram showing the five clusters of differentially expressed genes following HWB treatment. (A)** Plots of the expression profiles of 1,225 differentially expressed genes. Gray lines mark the various gene profiles; the green, red, blue, pink and light-blue lines represent the average expression profiles of clusters 1–5, respectively. **(B)** Heat map showing relative expression of 1,225 fruit genes at the four sampling times (0, 4, 17 and 48 h). Color key represents relative expression on a log 2 scale.

The expression patterns of 1,225 genes that were differentially expressed in at least one time point were subjected to hierarchical clustering, which resulted in five main clusters (Figure 4A), which then were visualized on a heat map (Figure 4B). The first cluster contained 343 genes, and clusters 2 through 5 contained 370, 120, 245, and 147 genes, respectively (Figures 4A,B). The expression patterns shown in Figures 4A and 4B indicate that, compared with their controls: cluster 1 showed increased transcript abundance at times 0 and 4 h and decreased abundance at times 17 and 48 h; cluster 2 was characterized by a transient increase in transcript abundance at time 0 h and decreases to almost no change at times 4, 17 and 48 h; cluster 3 showed transient up-regulation at 0 h and down-regulation at 17 h; cluster 4 was characterized by down-regulation of transcript abundance at 0 and 4 h, less marked down-regulation at 17 h, and almost returned to equal expression in treated and control fruits at 48 h after the treatment; cluster 5 showed an increased gene expression from time 0 to 4 which is maintained through 48 h.

**Table 1:** **Summary of differentially expressed genes following HWB treatment**

Time point after treatment (h)	Number of transcripts with increased abundance	Number of transcripts with decreased abundance	Total
0	99	728	827
4	141	195	336
17	137	38	175
48	17	70	87

### Analysis of over-represented gene ontology (GO) terms in the subset of differentially expressed genes relative to the mango transcriptome database

Each cluster was analyzed for its GO-enriched profile using BLAST2go and Fisher’s Exact Test (Additional file 5: Table S5). Cluster 1 (Figure 4A) was characterized by a transient increase in the expression of genes involved in defense against pathogens as well as in chitinase and amino-glycan activities (Additional file 5: Table S5). Genes that possibly are involved in inhibition of fungal growth by direct modulation of host structural changes as well as by induced host resistance were selected for further analysis [20]. This suggests that the HWB treatment induces fruit responses similar to those observed to be induced following pathogen invasion and during induction of transient stress resistance.

Cluster 2 were highly represented genes encoding for enzymes associated with flavanoid biosynthesis and metabolism, such as chalcone-flavanone isomerase family protein isoform 1 (EC:5.5.1.6) and flavonol synthase flavanone 3-hydroxylase-like (EC:1.14.11) [21]. Cluster 2 also included malic enzyme activity, such as NADP-dependent malic enzyme-like (EC:1.1.1.38), that catalyzes oxidative decarboxylation of malate to pyruvate [22].

Cluster 3 was enriched with genes associated with the cellular defense response and its regulation; in this cluster we found genes such as allene oxidase synthase (*AOS*) (EC:4.2.1.92) and syntaxin 121 (*Syn121*) [23].

Cluster 4 was enriched with genes involved in photosynthesis and chlorophyll catabolism, whose abundance decreased as sampling times increased from 0 through 17 h (Figure 3, Additional file 5: Table S5). Among the genes with reduced expression, encoding for protein that might contribute to the reduction of chlorophyll level in the fruit were chlorophyll a-b binding protein chloroplastic-like (*LHCIIb*) (EC4.99.1.1) [24, 25] and light-harvesting complex i protein (*lhca2*) [26], which resides in the chloroplast thylakoid membrane; also in this cluster was the gene photosystem i reaction center subunit chloroplast (*PIRC*) (EC1.97.1.12) [27]. Cluster 5 was not enriched with any GO classification term.

### Validation of differential gene expression

**Figure 5:**
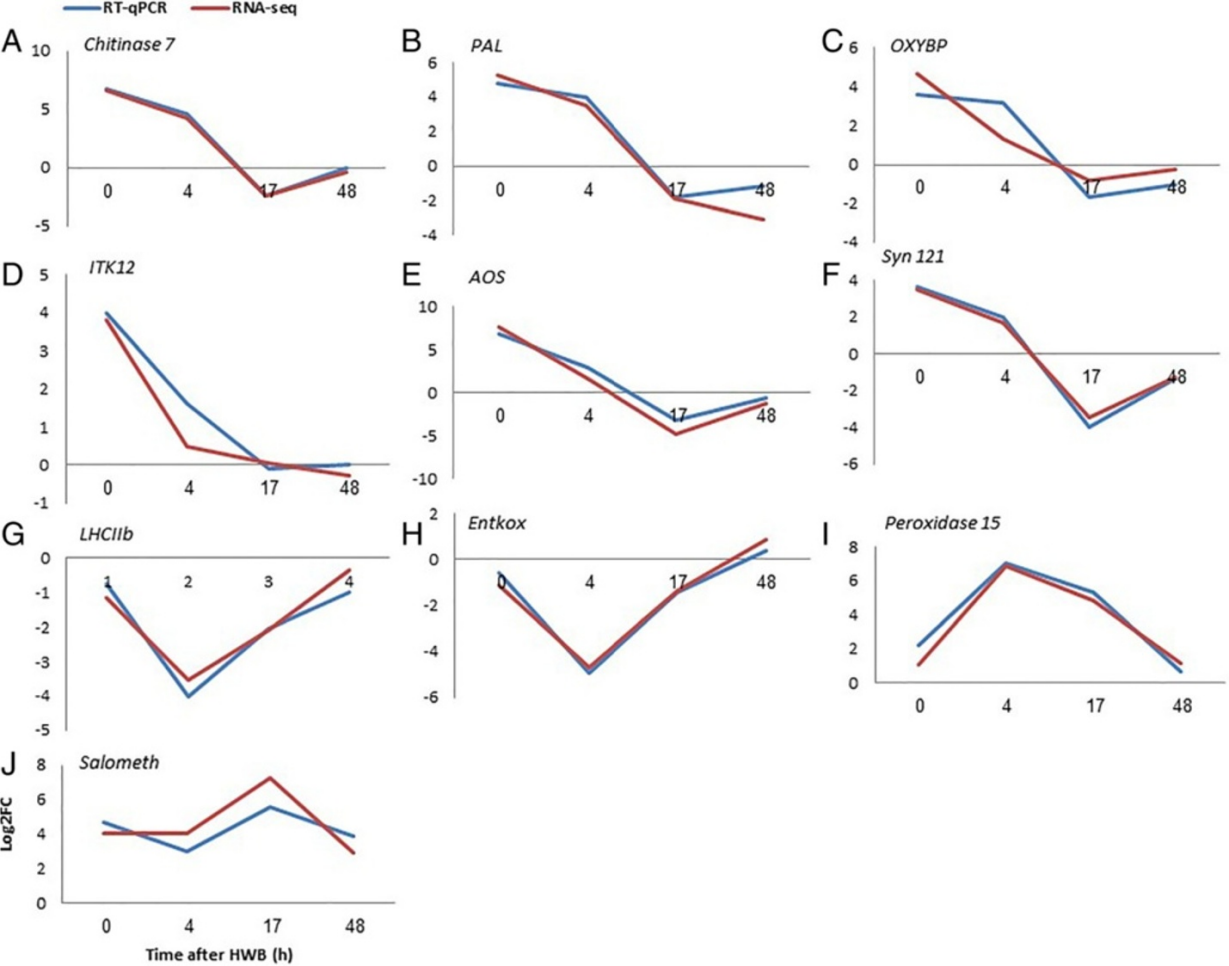
**Validation of RNA-seq results by means of qRT-PCR.** Ten differentially expressed genes (two from each of clusters 1 to 5) were examined by RNA-seq and qRT-PCR at four different time points after HWB treatment: **A**, **B** (cluster 1); **C**, **D** (cluster 2); **E**, **F** (cluster 3); **G**, **H** (cluster 4); and **I**, **J** (cluster 5). Values were normalized to the values obtained with untreated mango fruit samples at 0 h and the proportional fold-change (FC) was calculated. Expression data are means of two replicates.

To validate the differential expression of specific genes identified by the RNA-seq analysis, quantitative (q) RT-PCR analyses were performed for key genes of interest. Mango genes belonging to the five different clusters of genes that were differentially expressed following HWB treatment were analyzed for their expression levels (Figure 5). Chitinase 7 (EC:3.2.1.14) and phenylalanine ammonia lyase (*PAL*) (EC:4.3.1.25; EC:4.3.1.24) genes involved in chitinase activity and response to wounding were represented in cluster 1 (Figures 5A,B). Oxysterol binding protein (*OxyBP*) (EC:2.7.11.9) and inositol-tetrakisphosphate 1-kinase 2-like (*IT1K2*) (EC:2.7.1.159; EC:2.7.1.134) genes involved in jasmonic acid stimulus were represented in cluster 2 (Figure 5C,D). *AOS* and *Syn 121* genes involved in negative regulation of cellular defense responses were represented in cluster 3 (Figure 5E,F). *LHCIIb* and Ent-kaurene oxidase (*Entkox*) (EC:1.14.13.78) genes involved in photosynthesis and heme binding were represented in cluster 4 (Figure 5G,H). Salicylate o-methyltransferase (*salometh*) (EC 2.1.1.274) and peroxidase 15-like (*peroxidase15*) (EC 1.11.1.7) genes were represented in cluster 5 (Figure 5I,J). Comparison with the results of the qRT-PCR analysis showed expression patterns that were significantly and consistently similar to those of the RNA-seq analysis.

### The relationships among mango fruit HWB-induced responses, disease resistance to *A. alternata*and the differential expression of genes of the different clusters

**Figure 6:**
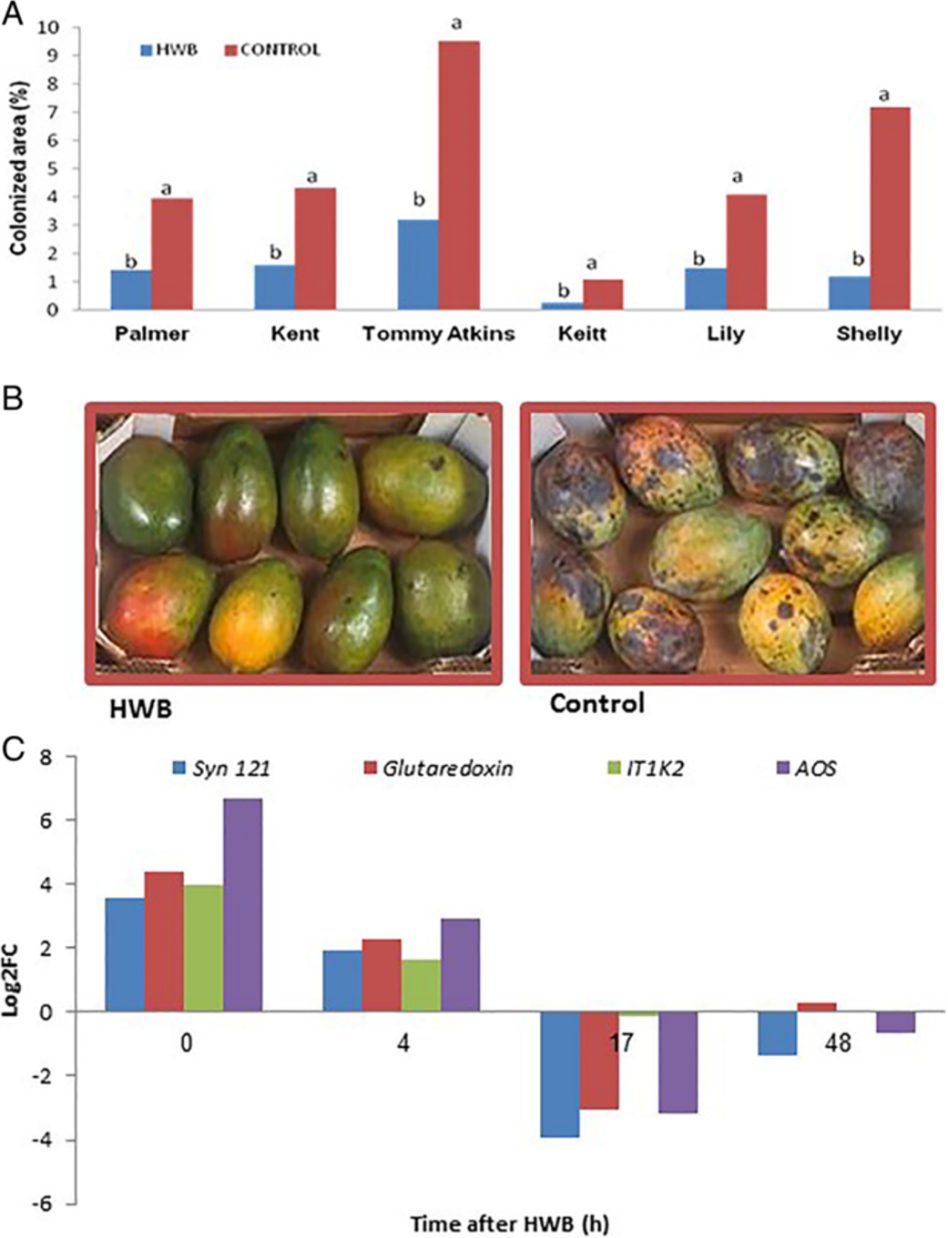
**Differential expressions of genes modulating the mechanism of resistance to** ***A. alternata*** **in naturally infected mango fruits. (A)** Effect of HWB on alternaria black spot (ABS) symptom development on mango cvs. Palmer, Kent, Tommy Atkins, Keitt, Lily and Shelly. **(B)** ABS symptom development on naturally infected fruits cv. Keitt following HWB treatment. **(C)** qRT-PCR differential expression profiling of genes *Syn121*, *glutardoxin*, *IT1K2* and *AOS* of cv. Shelly. Fruit peel tissues were sampled at four different time points after HWB treatment. RNA was extracted and served as a template for cDNA followed by qRT-PCR analysis of the genes of interest. Proportional increases in relative expression values were normalized against the samples of untreated mango fruits at 0 h. Expression data are means of two replicates. ABS-covered area was evaluated after 4 weeks of storage at 12°C. Average values followed by different letters differ significantly at *P* <0.05 according to the Tukey-Kramer HSD test.

HWB treatment of freshly harvested fruits reduced the incidence of natural *A. alternata* infestation on fruits of cvs. Palmer, Kent, Tommy Atkins, Keitt, Lilly and Shelly (Figure 6A). Hot water brushing treatment reduced the incidence of decay observed after 21 days of storage at 12°C by 64–84% (Figure 6A), as also observed in citrus [28–30] and in peaches [31]. Several genes (Figure 6C) that are known to modulate the host pathogen-resistance mechanism, related to JA and SA, showed upregulation [32–35]. *Syn121* gene showed significant differential expression (Figure 5F). This gene is a member of the SNARE protein family that contributes to defense against fungal penetration [36] and might be modulated by abiotic and biotic stress responses [37, 38]. It acts as a regulator of SA, and may contribute to host resistance in the fruit. SA contributes to systemic acquired resistance (SAR) [39] through processes that activate the hypersensitive reaction response and increased production of reactive oxygen species [40–42]. A second upregulated gene family in this group was that encoding *glutaredoxin* (EC 1.20.4.1). *Glutaredoxin* gene family is regarded as candidates for controlling the redox state of regulatory proteins [43]; they interact with TGA-transcription factors which are bZip plant transcription factors. These transcription factors contain a palindromic motif that is present in several plant promoters that are transcriptionally activated in response to elevated SA levels, and that negatively regulate the JA-inducible expression of defensin-like protein 16 (PDF1.2). *Gutaredoxin* is commonly used as a marker for JA-dependent defense responses [44].

Together with *Syn121*, *glutaredoxin* and *IT1K2* all have a significant effect on the jasmonic acid stimulus [45, 46] suggesting that the mechanism of induced resistance include JA-defense responses. Another major enzyme whose gene was upregulated was *AOS* (Figure 5E,F), which catalyzes the first step of conversion of linoleic acid to JA. A transient increase in *AOS* expression has been observed in wounded leaves [47] and it contributes to induced JA levels [48–50]. Changes in the metabolite content associated with the activities of ROS-scavenging enzymes were also detected in heated citrus fruits, indicating a similar possible major cellular reorganization process in those fruits, in response to the heat treatment [28, 29].

Aside from induction of host resistance, the presence of preformed antifungal alkylresorcinols such as resorcinol-5-(12-heptadecadienyl) and resorcinol-5-(pentadecyl) [14, 15] is also a key factor modulating fruit reisitance to postharvest pathogens. These compounds are fatty-acid derivatives obtained with specialized type III polyketide synthases (referred to as ‘alkylresorcinol synthases’), which catalyze the formation of 5-alkylresorcinols by using fatty acyl-CoA starter units and malonyl-CoA extension units. The polyketide synthase (PKS) enzymes involved in the biosynthesis of aromatic ring-containing intermediates as the resorcinol mainly use an aldol-condensation-based mechanism (stilbene synthase-type) or a Claisen-condensation-based mechanism (chalcone synthase-type) for ring folding [51]. A chalcone flavone isomerase involved in fatty-acid biosynthesis showed high expression at time 0 h after HWB, suggesting possible activation of this process by the inducing treatment.

### Effect of HWB on lenticel discoloration

**Figure 7:**
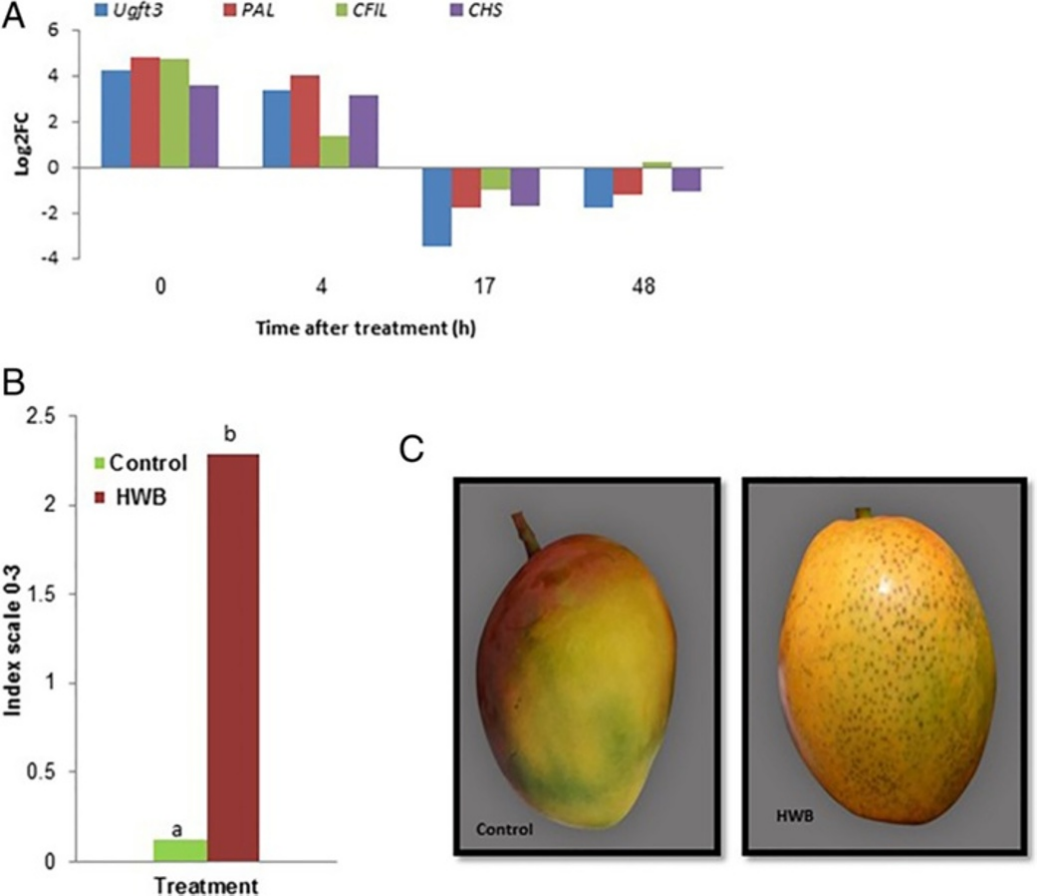
**Effects of HWB treatment on the expression of flavonoid biosynthesis-related genes and the occurrence of red lenticel discoloration on mango fruit cv. Shelly. (A)** qRT-PCR profile of differentially expressed genes *Ugft3, PAL, CFIL* and *CHSï*, which are related to the flavonoid biosynthesis process, naringenin-chalcone synthase activity, and the phenylpropanoid biosynthesis pathway. **(B)** level of lenticel discoloration of HWB-treated and control fruits, and **(C)** lenticel discoloration symptoms on mango fruits, cv. Shelly following HWB treatment. qRT-PCR values were normalized to the values obtained in samples from untreated mango fruits at 0 h. Expression data are means of two replicates. Lenticel discoloration was evaluated following 2 weeks of storage at 12°C [9]. Average values followed by different letters differ significantly at *P* <0.05 according to the Tukey-Kramer HSD test.

Lenticel discoloration results from stress induced by the HWB treatment, which leads to anthocyanin accumulation [9] (Figure 7). Four genes encoding UDP-glucose flavonoid 3-o-glucosyltransferase 3-like (*Ugft3*) (EC 2.4.1.91) – *PAL*, chalcone-flavanone isomerase-like protein (*CFIL*) (EC 5.5.1.6) and chalcone synthase (*CHS*) (EC:2.3.1.74) – related to the anthocyanin accumulation, phenylpropanoid and flavonoid biosynthesis pathways found in cluster 2 were tested by qRT-PCR (Figure 7A). Increased expression of these genes was clearly observed at the 0 and 4 h time points, followed by decreased expression at 17 and 48 h after the induction treatment. 1) *ugft3*, was described to control anthocyanin synthesis in grapes [52]; 2) *PAL*, encodes one of the major enzymes involved in flavonoid and phenylpropanoid biosynthesis in plants, and converts L-phenylalanine to ammonia and trans-cinnamic acid, which is the precursor of the polyphenol compounds [53, 54]; 3) *CFIL* is the second committed enzyme of the flavonoid biosynthetic pathway which enhances flavonoid production and pigmentation [55] 4) *CHS*, encoding chalcone synthase, which belongs to the PKSs and is also known as a type III PKS [56]; it catalyzes the initial step of flavonoid biosynthesis by converting 4-coumaroyl-CoA and malonyl-CoA to naringenin chalcone [57]. Although these genes peaked relatively early (4 h) after the HWB treatment, we envision that they activate relevant processes modulating fruit-resistance at later stages of fruit life. Similar phenylpropanoid pathway-expression pattern were described in heat treated peaches [58], suggesting a wide mechanism of fruit responses to heat treatment.

### Effect of HWB treatment on skin color change

**Figure 8:**
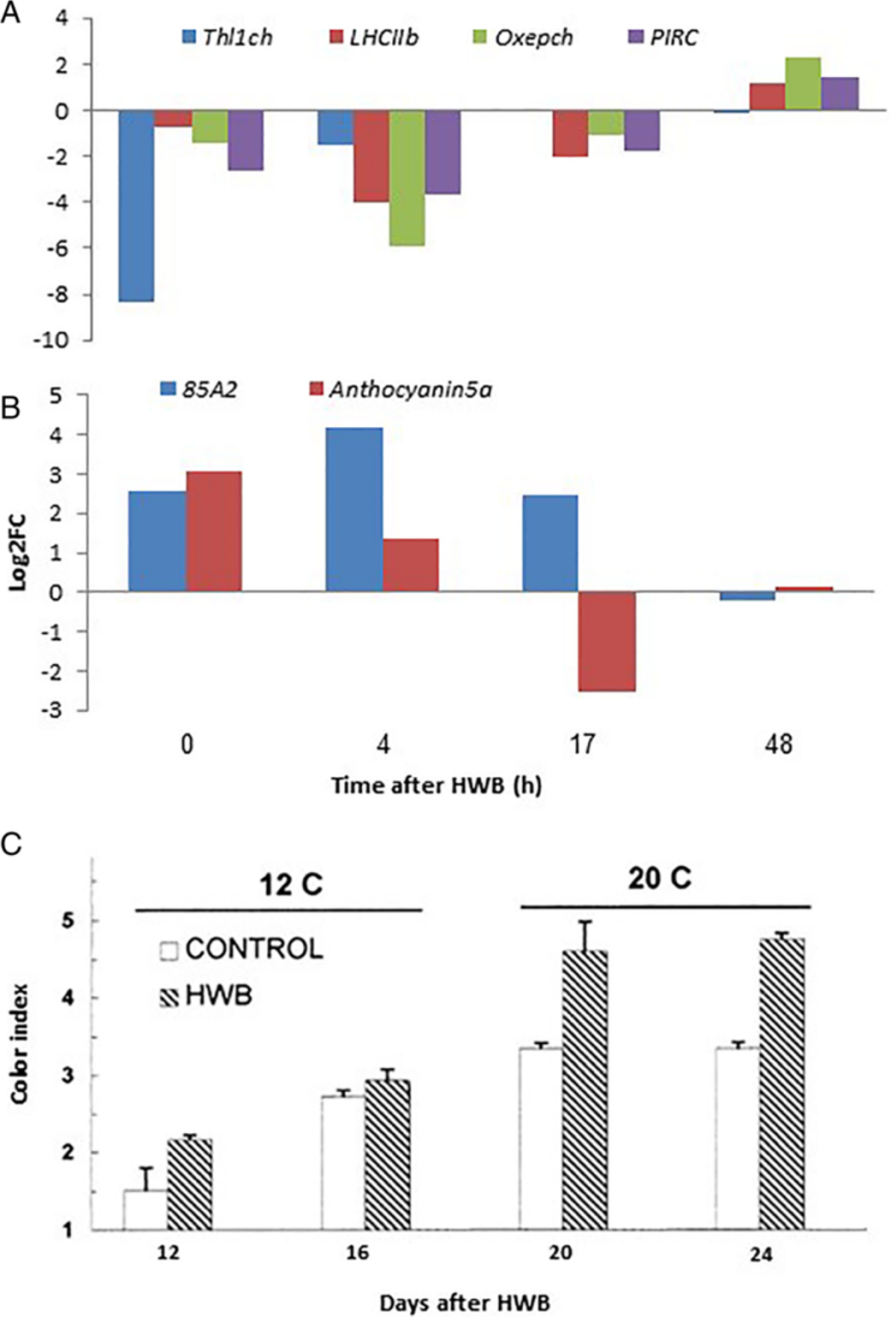
**Shelly. Effect of HWB on differential expression of chlorophyll and anthocyanin accumulation-related genes and color development in mango cv. Shelly. (A, B)** qRT-PCR gene-expression profiles of genes related to **(A)** chlorophyll accumulation (*Thl1ch*, *LHCIIb, Oxepch* and *PIRC*) and **(B)** anthocyanin synthesis (*85A2* and *Anthocyanin5*). The expression profile comprises data taken from samples of mango tissues sampled from cv. Shelly at four different time points after HWB treatment. **(C)** Changes in color index after 16 days of storage at 12°C followed by 8 days at 20°C. Vertical bars indicate SD of five replicates. qRT-PCR values were normalized to the values obtained with untreated mango fruit samples at 0 h. Expression data are the means of two replicates.

One of the significant consequences of HWB treatment is the improved and enhanced color development on the fruit skin, which results from both anthocyanin production and inhibition of chlorophyll accumulation. The fruit color index of HWB-treated fruits was higher during all the period of fruit storage at 12 and 20°C (Figure 8). The index in untreated fruits increased from values of 2.8, 16 days after harvest to 3.4, 8 days later. During the same period the HWB-treated fruits showed an increase in color index to 4.6 (Figure 8C), indicating induction of a 31% increase in color level by the HWB treatment. The decreased expression of chlorophyll and anthocyanin biosynthesis-related genes suggested that HWB played a role in the modulation of those processes (Figure 8A). Reduced expression of *LHCIIb* encoding for chlorophyll a/b-binding protein, one of the most abundant proteins in the chloroplast, which is important in the structure of photosynthesis reaction centers [24, 25, 59, 60] indicates a reduction in chlorophyll levels. Other key genes that showed reduced expression included those encoding: the oxygen-evolving enhancer protein chloroplastic (*Oxepch*) (EC1.3.1.74) [61]; *PIRC*; and thioredoxin-like 1-chloroplastic (*Thl1ch*) (EC 1.1.1.49), in which the chloroplast thioredoxins have been suggested as mediators in the light-dependent regulation of chloroplast enzyme activity [62].

Two genes related to anthocyanin accumulation – anthocyanin 5-aromatic (*anthocyanin5a*) (EC:2.3.1.144) and UDP-glycosyltransferase 85a2-like (*85A2*) (EC:2.4.1.115) – showed increased expression at the first two time points (0 and 4 h) after HWB, as detected by qRT-PCR (Figure 8B). The *anthocyanin5a* modulates anthocyanin by aromatic acylation [63] and *85A2* affects the level of glucosyl anthocyanidins in red fruit during ripening [52]. This suggests that different pathways are activated by HWB to modulate anthocyanin accumulation and to reduce processes associated with chlorophyll accumulation [64], thereby enhancing color development.

## Conclusions

Overall, gene-expression profiling in mango skin revealed similarities in heat responses to those found in citrus and peach fruits [30, 58], with three major effects following HWB treatment: 1) a transient increase in expression of the stress- and pathogen-defense mechanisms-related genes; 2) a transient reduction in the expression of chlorophyll-related genes; and 3) increased expression of sugar and flavonoid metabolism related genes 4 h after treatment. These three main trends indicated by the observed HWB-induced modulation of gene expression can account for the major results of postharvest HWB treatment including: 1) induced resistance to *A. alternata*, attributed to the transient increase in the expression of genes involved in immune response and host resistance (Figure 6); 2) improved color development observed after HWB which can be attributed to decreased photosynthesis, including reduction of chlorophyll accumulation after the treatment (Figure 8) and increased abundance of genes of flavonoid metabolism; and 3) enhanced lenticel discoloration that is also correlated with upregulation of flavanoid metabolism (Figure 7).

In light of the physiological changes described, the improved fruit quality acquired following heat stress is probably a result of various stress-response mechanisms that act coordinately to improve the fruit quality, prevent pathogen development, prevent cell damage and re-establish cellular homeostasis. Genes identified in the present study that are modified in mango fruits following heat treatment, could have similar functions in other fruits such as citrus or peach.

## Methods

### Fruit, and storage conditions

Freshly harvested mango fruits (*Mangifera indica* L. cv. Shelly) were obtained from trees in commercial orchards in the north of Israel. Several experiments to determine the phenotypic response of the fruits to HWB were carried out in each season during three consecutive years. The phenotypic responses obtained in the various years were similar, therefore results from only 1 year are presented. Each treatment comprised 6 replications, each with 15 fruits.

Fruits were harvested at the commercial mature-green stage, treated on the same or the following day, and transferred to simulated export conditions. They were stored for about 4 weeks (depending on the experiment) at 12°C, ca. 90% RH [9]. Control, untreated fruits were stored under the same conditions, immediately upon arrival from the orchard.

### Postharvest packinghouse treatments in semi-commercial mango experiments

Postharvest treatments were carried out at the Department of Postharvest Science of the Volcani Center in Bet Dagan, Israel. HWB treatment at 55°C was applied on the packing line, as a spray (nozzle pressure of 2 atm) above brushes revolving at 60 g, a rate of 100–120 L min^-1^ and at a nozzle pressure of 2 atm [2]. Fruits were passed over five to seven transversely oriented, 12-cm-diameter plastic brushes for 15–20 s.

### Fruit ripening, development of red lenticels, black spot and stem-end rots during storage

Disease severity, measured as the percentage of the fruit surface covered by black lesions, was recorded for 90 fruits (15 fruits from each of six replicates) after about 4 weeks of storage at 12°C and 3 days at 20°C. Fruits were regarded as unmarketable when more than 1% of their surface area exhibited black spots.

Lenticel spotting was assessed visually at the end of storage, on a scale of 0 to 3, with values based on a combination of degree of lenticel discoloration and area covered by the symptoms [9].

Skin color development was assessed visually at the end of storage and a color index was calculated on a scale of 1 (green) to 5 (full color) [9].

### Total RNA extraction

Total RNA was extracted according to Yang et al. [65], with minor changes: about 1- to 2-g aliquots of HWB-treated and control tissues were sampled from pools composed of five different fruits from the same tree. The samples were ground to a fine powder in liquid nitrogen and transferred into 50-mL centrifuge tubes with 10 mL of CTAB RNA extraction buffer (100 mM Tris-borate pH 8, 2 M NaCl, 25 mM ethylenediaminetetraacetic acid pH 8, 2% (w/v) CTAB, 2% (w/v) polyvinylpolypyrrolidone and 2% (v/v) β-mercaptoethanol). The mixture was shaken for 3 min and then incubated at 65°C for 15 min. Samples were extracted twice with an equal volume of chloroform:isoamyl alcohol [24:1 (v/v)], and the phases were separated by centrifugation at 10,000 g for 10 min. Following centrifugation, lithium chloride was added to a final concentration of 2.5 M and RNA was allowed to precipitate overnight at 4°C. RNA was pelleted at 4°C for 30 min at 10,000 *g*, washed with 70% ethanol, and re-suspended at 65°C for 3 min in SSTE buffer (10 mM Tris pH 8, 1 M NaCl, 1 mM EDTA pH 8 and 0.5% (w/v) SDS). Samples were extracted with an equal volume of chloroform:isoamyl alcohol (24:1), and with an equal volume of chloroform:isoamyl alcohol:water-saturated phenol (24:1:25), and the phases were separated by centrifugation at 10,000 g for 10 min. The RNA was ethanol-precipitated overnight, and resuspended in diethyl-pyrocarbonate-treated water. RNA was further treated with Turbo DNAse (Ambion, Austin, TX, USA).

### Analysis by qRT-PCR

Single-stranded cDNA was synthesized from 1 μg of total RNA by means of the Verso cDNA synthesis kit (Thermo Fisher Scientific, Waltham, MA, USA). The synthesized cDNA was used as a template for qRT-PCR analysis, to estimate the expression level of the selected genes. The cDNA samples were diluted 1:10 (v/v) to the final template concentration for qRT-PCR. Real-time qRT-PCR was performed with a RotorGene 3000 system (Corbett Research, Sydney, Australia). PCR amplification was run with 3.5 μL of cDNA template in 10 μL of reaction mixture containing 5 μL absolute blue qPCR SYBR green ROX mix (Thermo Scientific) and 300 nM primers. PCR conditions were: initial denaturing for 15 min at 94°C; 40 denaturing cycles of 10 s at 94°C; annealing at 60°C for 15 s; extension at 72°C for 20 s (cycling A), 77°C for 6 s (cycling B), or 80°C for 6 s (cycling C), and melting at 72–99°C. The samples were subjected to melting-curve analysis with the RotorGene program. All samples were normalized to actin gene levels in the same qRT-PCR, and the values were expressed as increase or decrease in level relative to a calibration sample. The forward and reverse primers for all of the genes are listed in Table 2.

**Table 2:** **Primers used for qRT-PCR analysis**

Gene	Forward 5‘ → 3’	Reverse 5‘ → 3’
*Actin*	CATTGTGCTCAGTGGTGGTT	TTGGAGCAAGTGCAGTGATT
*FSL*	TGGGAGCATATGTTAGGGTATTGG	TCTCATCGCCTCATAATTCAAGAA
*PAL*	AATGGAAATGCGGCGATTC	TCCCAGCTCTTCCCTCACAA
*CHS*	GAAAGATGTTCCCGGGTTGA	AGTCGTTGATGCCGATTGG
*CFIL*	GCAGTGATCCCACCACTTGA	TCATGCATTCGTACATCTGTAGCA
*Oxybp*	CAATTCGACGGAACCGAATC	CCGTTCCCTACCAAGTCGTTT
*IT1K2*	AACTATTTCCCTGGGTATGGGAA	CTGACTCTGTTTCAGGCCCAA
*Chitinase 7*	CATTCAAGACTGCCCTGTGGTA	GGCTCGAATAGTTGCACCAAA
*Hl1ch*	TGCAACACCAAAGCAACAAAC	TCCCACCATCTAAGAGACCTACTG
*PIRCS*	AGTCTAACGCTGACAGGAAGGAA	AATCCACCAGCGAACTTGGA
*LHCIIb*	GGGCTATGCTTGCTGTTCCA	AGCCCACTCTTGAGCCTTTACC
*85A2*	CAAGCTTCATGAGAGTCACTGATG	GAAGATTTCAAGCAGTCGTGTGAT
*Entkox*	TGGCAGTGAGATTGCCGTAA	CAGGCCTCCACACGTTAGGAT
*Salometh*	ACTCACCATCACCAAGCGAAA	TTGTGTAATCTCAGTCCGATCAATC
*Syn121*	TGCGGTCCAGGATCTTCATC	TCCATCGAGTCCTGCAACTTC
*Glutaredoxin*	GGGAAGATGGTTTCCGAGAAT	AGCAAACGCTTCACCACATG
*AOS*	AGAGCAGAGGAGTTCGTGGC	TGGTCCGTTTGACCACAGC
*Peroxidase15*	TGCCCAGGAGTTGTCTCTTGT	CAGGTTTGACCTCCATCCAAA
*Ugft3*	CTCATCTGCAATCCAGAAATCG	AATCGAGTGTCGCGGTTTG
*Thl1ch*	TGCAACACCAAAGCAACAAAC	TCCCACCATCTAAGAGACCTACTG
*Oxepch*	GCCACGTCTCGAACATTAAGC	TACACTGCCGTCGTCTTCTTGA
*Anthocyanin5*	TTTCTCACTTCCCTGCTTTGG	TCCGGCGTCATTACAGATGA

### Bioinformatics analysis of RNA-seq data

The transcriptome of *M. indica* cv. Shelly was sequenced according to Illumina Hiseq2000 and Trueseq protocols, at the Crown Institute for Genomics, The Nancy and Stephen Grand Israel National Center for Personalized Medicine at the Weizmann Institute of Science, Rehovot, Israel. Eight libraries with total single-end RNA-seq reads 100 nucleotides in length were generated. The eight libraries contained the following sequences: 1) control RNA-seq of mango peel at time 0 h with 20,025,080 reads; 2) RNA-seq of mango peel treated by HWB at time 0 h with 20,202,891 reads; 3) control RNA-seq of mango peel at time 4 h with 21,622,332 reads; 4) RNA-seq of mango peel 4 h after HWB with 20,744,762 reads; 5) control RNA-seq of mango peel at 17 h with 20,916,444 reads; 6) RNA-seq of mango peel 17 h after HWB with 21,410,839 reads; 7) control RNA-seq of mango peel at 48 h with 20,687,358 reads; and 8) RNA-seq of mango peel 48 h after HWB with 21,230,696 reads. The transcriptome datasets are available in the NCBI Sequence Read Archive (SRA) under accession number SRX375390 and BioProject accession PRJNA227243. A new transcriptome was assembled from the 8.6-Gbp sequences by using Trinity software [66], generating 57.544 contigs with N50 of 1,598 bp. This Transcriptome Shotgun Assembly project has been deposited at DDBJ/EMBL/GenBank under accession no. GBCV00000000. The version described in the present paper is the first version, GBCV01000000.

Tophat [67], Bowtie2 [68] and Cufflink packages [67] were used to align the RNA-seq with the transcriptome and to calculate differentially expressed genes. The libraries were aligned with the mango transcriptome at alignment rates (mapped reads/total reads) of 90.84, 90.02, 89.48, 89.77, 90.70, 90.11, 90.25 and 90.35% for samples 1 to 8, respectively.

The genes of *M. indica* cv. Shelly were annotated by using BLASTx [69], after which their GO term [70] was assigned by combining both BLASTx data and interproscan analysis [71] by means of the BLAST2go software pipeline [72]. GO-enrichment analysis was performed by using Fisher’s exact test with multiple testing correction of FDR. Heat mapping and clustering of the genes were performed with the R software ggplots2 package [73].

## Electronic supplementary material

Additional file 1: Table S1: Table of differentially expressed genes and their annotations after HWB treatment versus control at time 0 h. (XLSX 5 MB)

Additional file 2: Table S2: Table of differentially expressed genes and their annotations after HWB treatment versus control at time 4 h. (XLSX 5 MB)

Additional file 3: Table S3: Table of differentially expressed genes and their annotations after HWB treatment versus control at time 17 h. (XLSX 5 MB)

Additional file 4: Table S4: Table of differentially expressed genes and their annotations after HWB treatment versus control at time 48 h. (XLSX 5 MB)

Additional file 5: Table S5: GO enrichment analysis of gene clusters [74]. (DOC 100 KB)

## Abbreviations

HWB: Hot-water brushing
*OxyBP*: Oxysterol-binding protein
*IT1K2*: Inositol-tetrakisphosphate 1-kinase 2-like
*AOS*: Allene oxide synthase
*Syn121*: Syntaxin-121-like
*LHCIIb*: Chlorophyll a/b-binding protein chloroplastic-like
*PIRCS*: Photosystem I reaction center subunit chloroplastic-like
*85A2*: UDP-glycosyltransferase 85a2-like
*PAL*: Phenylalanine ammonia lyase
*CFIL*: Chalcone-flavanone isomerase-like protein
*CHS*: Chalcone synthase
*Entkox*: Ent-kaurene oxidase
*Salometh*: Salicylate o-methyltransferase
Ugft3: UDP-glucose flavonoid 3-o-glucosyltransferase
*Thl1ch*: Thioredoxin-like 1- chloroplastic
*Oxepch*: Oxygen-evolving enhancer protein chloroplastic.
