# Supplementary material for: De-novo assembly of mango fruit peel transcriptome reveals mechanisms of mango response to hot water treatment
Source: BMC Genomics. 2014 Nov 5;15(1):957. doi: 10.1186/1471-2164-15-957 (PMC4236434; doi:10.1186/1471-2164-15-957)
Supplement: Supplementary file 5 — Additional file 5: Table S5: GO enrichment analysis of gene clusters [74]. (DOC 100 KB) [file 12864_2014_6661_MOESM5_ESM.doc]

**Table 2: GO enrichment analysis of gene clusters**

| Cluster color | GO-ID | Term | Category* | FDR** | *P*-Value |
| --- | --- | --- | --- | --- | --- |
| 1 | GO:0008061 | chitin binding | F | 8.38E-08 | 1.26E-11 |
|  | GO:1901072 | glucosamine-containing compound catabolic process | P | 2.24E-05 | 3.03E-08 |
|  | GO:0006032 | chitin catabolic process | P | 2.24E-05 | 3.03E-08 |
|  | GO:0006030 | chitin metabolic process | P | 2.24E-05 | 3.03E-08 |
|  | GO:0006026 | aminoglycan catabolic process | P | 2.24E-05 | 3.03E-08 |
|  | GO:0046348 | amino sugar catabolic process | P | 2.24E-05 | 3.03E-08 |
|  | GO:0004568 | chitinase activity | F | 2.37E-05 | 4.07E-08 |
|  | GO:0003700 | sequence-specific DNA binding transcription factor activity | F | 2.37E-05 | 4.64E-08 |
|  | GO:0001071 | nucleic acid binding transcription factor activity | F | 2.37E-05 | 4.64E-08 |
|  | GO:0006022 | aminoglycan metabolic process | P | 2.41E-05 | 5.39E-08 |
|  | GO:0009620 | response to fungus | P | 2.41E-05 | 5.44E-08 |
|  | GO:1901071 | glucosamine-containing compound metabolic process | P | 3.77E-05 | 9.08E-08 |
|  | GO:0006040 | amino sugar metabolic process | P | 1.09E-04 | 2.80E-07 |
|  | GO:0009055 | electron carrier activity | F | 5.30E-04 | 1.60E-06 |
|  | GO:0050832 | defense response to fungus | P | 6.15E-04 | 1.95E-06 |
|  | GO:0043900 | regulation of multi-organism process | P | 2.71E-03 | 1.02E-05 |
|  | GO:0016998 | cell wall macromolecule catabolic process | P | 3.04E-03 | 1.19E-05 |
|  | GO:0010200 | response to chitin | P | 3.35E-03 | 1.36E-05 |
|  | GO:1901700 | response to oxygen-containing compound | P | 4.11E-03 | 1.86E-05 |
|  | GO:0004553 | hydrolase activity, hydrolyzing O-glycosyl compounds | F | 5.10E-03 | 2.38E-05 |
|  | GO:0010243 | response to organonitrogen compound | P | 6.52E-03 | 3.14E-05 |
|  | GO:0003677 | DNA binding | F | 8.05E-03 | 4.22E-05 |
|  | GO:0005509 | calcium ion binding | F | 8.80E-03 | 4.77E-05 |
|  | GO:0009607 | response to biotic stimulus | P | 9.99E-03 | 5.57E-05 |
|  | GO:0016798 | hydrolase activity, acting on glycosyl bonds | F | 1.22E-02 | 6.96E-05 |
|  | GO:0000249 | C-22 sterol desaturase activity | F | 1.30E-02 | 7.86E-05 |
|  | GO:0050691 | regulation of host defense response to virus | P | 1.30E-02 | 7.86E-05 |
|  | GO:2001141 | regulation of RNA biosynthesis process | P | 1.46E-02 | 9.24E-05 |
|  | GO:0006355 | regulation of DNA-dependent transcription | P | 1.46E-02 | 9.24E-05 |
|  | GO:0051252 | regulation of RNA metabolic process | P | 1.52E-02 | 9.83E-05 |
|  | GO:0051707 | response to other organisms | P | 1.81E-02 | 1.21E-04 |
|  | GO:0019438 | aromatic compound biosynthesis process | P | 1.81E-02 | 1.23E-04 |
|  | GO:0005506 | iron ion binding | F | 1.82E-02 | 1.26E-04 |
|  | GO:0009611 | response to wounding | P | 2.03E-02 | 1.43E-04 |
|  | GO:0010033 | response to organic substance | P | 2.52E-02 | 1.82E-04 |
|  | GO:0042537 | benzene-containing compound metabolic process | P | 2.64E-02 | 1.95E-04 |
|  | GO:1901362 | organic cyclic compound biosynthesis process | P | 2.93E-02 | 2.24E-04 |
|  | GO:0016491 | oxidoreductase activity | F | 2.93E-02 | 2.29E-04 |
|  | GO:0070704 | sterol desaturase activity | F | 2.93E-02 | 2.34E-04 |
|  | GO:0010262 | somatic embryogenesis | P | 2.93E-02 | 2.34E-04 |
|  | GO:0010556 | regulation of macromolecule biosynthesis process | P | 4.07E-02 | 3.52E-04 |
|  | GO:2000112 | regulation of cellular macromolecule biosynthesis process | P | 4.07E-02 | 3.52E-04 |
|  | GO:0031347 | regulation of defense response | P | 4.07E-02 | 3.56E-04 |
|  | GO:0031326 | regulation of cellular biosynthesis process | P | 4.42E-02 | 4.06E-04 |
|  | GO:0002697 | regulation of immune effector process | P | 4.91E-02 | 4.66E-04 |
|  | GO:0050688 | regulation of defense response to virus | P | 4.91E-02 | 4.66E-04 |
|  | GO:0003700 | sequence-specific DNA binding transcription factor activity | F | 2.25E-04 | 6.78E-08 |
|  | GO:0001071 | nucleic acid binding transcription factor activity | F | 2.25E-04 | 6.78E-08 |
|  | GO:0005388 | calcium-transporting ATPase activity | F | 1.51E-03 | 6.81E-07 |
|  | GO:0070588 | calcium ion transmembrane transport | P | 1.80E-03 | 1.09E-06 |
|  | GO:0016682 | oxidoreductase activity, acting on diphenols and related substances as donors; oxygen as acceptor | F | 2.88E-03 | 2.17E-06 |
|  | GO:0015085 | calcium ion transmembrane transporter activity | F | 7.03E-03 | 6.36E-06 |
|  | GO:0016679 | oxidoreductase activity, acting on diphenols and related substances as donors | F | 8.70E-03 | 1.16E-05 |
|  | GO:0004674 | protein serine/threonine kinase activity | F | 8.70E-03 | 1.18E-05 |
|  | GO:0015662 | ATPase activity, coupled to transmembrane movement of ions, phosphorylative mechanism | F | 1.44E-02 | 2.39E-05 |
| *2* | GO:0004672 | protein kinase activity | F | 2.86E-02 | 6.39E-05 |
|  | GO:0016619 | malate dehydrogenase (oxaloacetate-decarboxylating) activity | F | 2.86E-02 | 6.53E-05 |
|  | GO:0004470 | malic enzyme activity | F | 2.86E-02 | 6.53E-05 |
|  | GO:0052716 | hydroquinone:oxygen oxidoreductase activity | F | 2.86E-02 | 6.53E-05 |
|  | GO:0015398 | high-affinity secondary active ammonium transmembrane transporter activity | F | 2.86E-02 | 6.89E-05 |
|  | GO:0008447 | L-ascorbate oxidase activity | F | 3.12E-02 | 8.92E-05 |
|  | GO:0046274 | lignin catabolic process | P | 3.12E-02 | 8.92E-05 |
|  | GO:0046271 | phenylpropanoid catabolic process | P | 3.12E-02 | 8.92E-05 |
| 3 | GO:0051245 | negative regulation of cellular defense response | P | 1.57E-02 | 4.74E-06 |
|  | GO:0043495 | protein anchor | F | 1.57E-02 | 4.74E-06 |
|  | GO:0006968 | cellular defense response | P | 2.36E-02 | 1.42E-05 |
|  | GO:0010185 | regulation of cellular defense response | P | 2.36E-02 | 1.42E-05 |
| 4 | GO:0009765 | photosynthesis, light harvesting | P | 2.72E-04 | 4.10E-08 |
|  | GO:0046906 | tetrapyrrole binding | F | 1.76E-03 | 5.31E-07 |
|  | GO:0015979 | photosynthesis | P | 3.18E-03 | 1.44E-06 |
|  | GO:0020037 | heme binding | F | 3.38E-03 | 2.04E-06 |

*Category – Describes the GO category – F-indicate Molecular Function; P-indicate biological process; and C- indicate Cellular Component

**FDR – false discovery rate –Benjamini and Hochberg .
